# Assessing the Occurrence and Distribution of Microplastics in Surface Freshwater and Wastewaters of Latvia and Lithuania

**DOI:** 10.3390/toxics11040292

**Published:** 2023-03-23

**Authors:** Reza Pashaei, Viktorija Sabaliauskaitė, Sergej Suzdalev, Arūnas Balčiūnas, Ieva Putna-Nimane, Robert M. Rees, Reda Dzingelevičienė

**Affiliations:** 1 Marine Research Institute, Klaipeda University, 92294 Klaipeda, Lithuania; 2 Latvian Institute of Aquatic Ecology, 1007 Riga, Latvia; 3 Scotland’s Rural College (SRUC), West Mains Road, Edinburgh EH9 3JG, UK; 4 Faculty of Health Sciences, Marine Research Institute, Klaipeda University, 92294 Klaipeda, Lithuania

**Keywords:** microplastics, occurrence, distribution, surface water, wastewaters

## Abstract

Microplastic concentrations in surface water and wastewater collected from Daugavpils and Liepaja cities in Latvia, as well as Klaipeda and Siauliai cities in Lithuania, were measured in July and December 2021. Using optical microscopy, polymer composition was characterized using micro-Raman spectroscopy. The average abundance of microplastics in surface water and wastewater samples was 16.63 ± 20.29 particles/L. The dominant shape group of microplastics in water was fiber, with dominant colors found to be blue (61%), black (36%), and red (3%) in Latvia. Similar distribution in Lithuania was found, i.e., fiber (95%) and fragments (5%) with dominant colors, such as blue (53%), black (30%), red (9%), yellow (5%), and transparent (3%). The micro-Raman spectroscopy spectra of visible microplastics were identified to be polyethylene terephthalate (33%) and polyvinyl chloride (33%), nylon (12%), polyester (PS) (11%), and high-density polyethylene (11%). In the study area, municipal and hospital wastewater from catchment areas were the main reasons for the contamination of microplastics in the surface water and wastewater of Latvia and Lithuania. It is possible to reduce pollution loads by implementing measures such as raising awareness, installing more high-tech wastewater treatment plants, and reducing plastic use.

## 1. Introduction

The emergence of plastic as a major component of modern life began in the 1950s and has grown exponentially ever since. Plastic production and consumption are estimated to have reached 368 million tons in 2019, representing an increase of over 200% from the pre-1950s era [1]. As a result, plastic has become an omnipresent component of our lives and is found in nearly every household and commercial space. Such widespread use and disposal of plastics have raised significant environmental concerns, such as plastic pollution, which have become global issues in recent years. Plastics are often preferred to other materials due to their durability, malleability, low cost, versatility, and impermeability. However, concerns are increasingly being raised about the persistence and accumulation of plastics in the environment [2,3]. An increase in environmental concentrations of plastics is expected due to increased demand and production and a lack of adequate waste processing capacity [4]. Microplastic (MPs) in the aquatic environment originate from intentional and unintentional losses of plastics and MPs, such as littering, loss of fishing gear, loss of granules used for manufacture, and release in wastewater effluents [5,6,7]. In order to be more effective at removing MPs from the environment, advanced techniques must be developed for wastewater treatment plants (WWTPs) [8]. Several studies have been conducted on microplastic (MP) pollution in the Baltic Sea and adjacent countries, showing high rates of MP pollution [9,10,11,12,13,14,15,16,17,18,19].

The Baltic Sea is one of the world’s largest brackish water bodies and includes the Kattegat, which is home to 6065 species overall, including 1700 phytoplankton, 442 phytobenthos, 1199 zooplankton, 569 meiozoobenthos, 1476 macrozoobenthos, 380 vertebrate parasites, 200 fish, three seal species, and 83 bird species [20]. This richness in species diversity highlights the sensitivity of the environment to pollution and the need for more knowledge and understanding of the prevalence and behavior of MPs in the region.

## 2. Materials and Methods

### 2.1. Sample Collection and Sample Preparation

**Figure 1:**
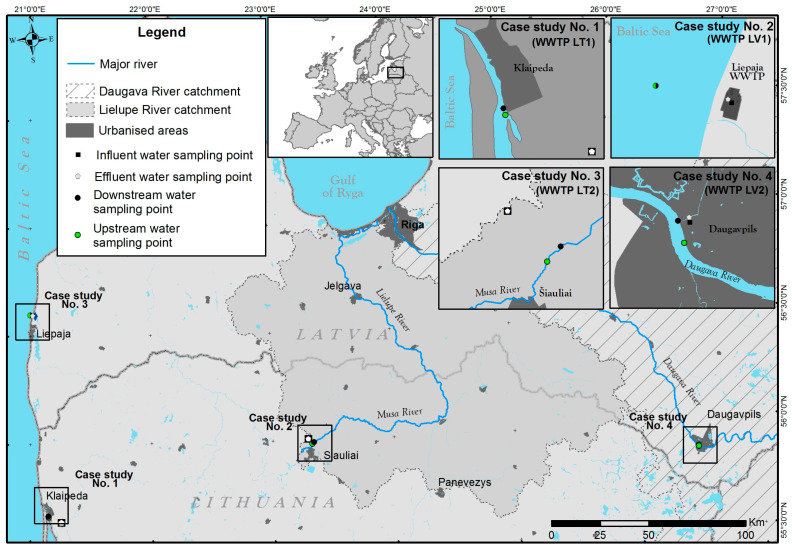
Sampling stations of Latvia and Lithuania.

In Latvia and Lithuania, a comprehensive sampling campaign was conducted in July and December 2021 to evaluate the water quality in various locations within each city. In Daugavpils and Liepaja (Latvia) and Klaipeda and Siauliai (Lithuania), a total of 32 water samples were collected from four different sites in each city, including influent, effluent, upstream, and downstream locations (Lithuania). Before and after treatment, influent (n = 8) and effluent (n = 8) samples were collected at each sampling site. Following wastewater treatment, 16 samples of background surface water from upstream (n = 8) and downstream (n = 8) points were collected at a distance of 500 m from the discharge point. Notably, the upstream and downstream samples (SLV3/WLV3, SLV4/WLV4) of Liepaja were collected in the Baltic Sea, above the wastewater discharge point, where brackish water is present. Indeed, four samples were collected from each city in July and December. Figure 1 and Table S1 provide additional information on sampling locations and methods.

### 2.2. Sample Preparation

In July and December 2021, 200 mL samples were collected from the influent, the effluent, the upstream, and the downstream. The pH, temperature, and dissolved oxygen of the samples were measured at the time of collection using the Multi 9630 IDS, WTW, ODO200, EcoSense, and ODO200, EcoSense instruments, respectively (Table S1). The water samples were stored at −20 °C in appropriate storage containers that were clean, dry, and properly labeled with the date and location of the sample. After that, the samples were removed from storage and allowed to come to room temperature. 20 mL of hydrogen peroxide solution 30% (Merck, Germany) was added to each sample bottle and shaken for 2 h at room temperature. The samples were filtered through a pre-weighed 0.45 μm, 47 mm white-gridded mixed cellulose-ester membrane filter (Frisenette, Denmark).

### 2.3. Laboratory Analysis

#### 2.3.1. Optical Microscopy Analysis of the Microplastics

Analysis via optical microscopy is a common technique for examining MPs in water samples. However, it may not always be possible to differentiate plastic particles from other organic or inorganic materials based solely on their morphological characteristics. To confirm the identity of the particles, additional analytical methods, such as micro-Raman spectroscopy, are required. In our study, we analyzed the quantity, color, shape, and size of the suspected MPs using optical microscopy as the initial step in plastic screening for all samples. Using optical microscopy, small fragments of shells and other materials may be misidentified as MPs. We used micro-Raman spectroscopy to detect and confirm the presence of MP particles in the water samples in order to address this issue. We utilized a Nikon SMZ800N stereo microscope with a camera and an Olympus KL 1500 LCD microscope to directly examine filter samples obtained in situ. The particle sizes were classified into four categories: <0.25 mm, 0.25–0.5 mm, 0.5–1 mm, and 1–5 mm.

Optical microscopy is a reliable technique for detecting and analyzing MPs because it permits the visualization and characterization of the particles at relatively high magnification. Notably, optical microscopy has limitations when it comes to the detection and analysis of MPs. Optical microscopy is only able to detect MPs that are large enough to be resolved by the objective lens. This means that optical microscopy may not be able to detect MPs that are smaller than 50 μm in size. Optical microscopy can only detect transparent or semi-transparent MPs due to the technique’s reliance on light transmission through the sample. MPs that are opaque, such as those that are black or extremely dark in color, may not be detectable with optical microscopy. In addition, certain types of MPs, such as those composed of particular plastics or coated with particular substances, may be difficult to visualize using optical microscopy due to the way in which they scatter light. Overall, while optical microscopy can be a useful tool for detecting and analyzing MPs, it is important to consider its limitations and to use other techniques, such as Raman spectroscopy, in conjunction with optical microscopy in order to obtain a complete understanding of the MPs in a sample.

#### 2.3.2. Micro-Raman Spectroscopy Analysis of the Microplastics

Visual observation can detect millimeter-sized plastic particles, but identifying the specific type of plastic requires advanced spectroscopic techniques, such as Fourier transform infrared spectroscopy, micro-Raman spectroscopy, optical microscopy, and scanning electron microscopy in combination with energy dispersive X-ray spectroscopy [21,22]. These techniques enable a detailed analysis of the chemical composition and structural properties of MPs, allowing for a greater comprehension of their environmental impact. Micro-Raman is used to determine the type of polymer and to specify the particle size and distribution of MP particles [23]. Specifically, the Raman technique is gaining ground rapidly in the analysis of MPs due to its higher spatial resolution (1 μm), broader spectral coverage, greater sensitivity to non-polar functional groups, lower water interference, and narrower spectral bands [24]. In addition, micro-Raman is an indispensable tool for the analysis of very small MPs (<20 μm) [25]. The micro-Raman technique is a powerful analytical tool that can be used to identify and characterize materials. It is a non-destructive technique that can be used to analyze a wide range of materials, including polymers, metals, semiconductors, and biological samples. Micro-Raman spectroscopy is an effective and efficient technique for detecting and identifying MPs in water samples. It is a non-destructive, sensitive, fast, inexpensive, and safe technique that can be used in a variety of settings. The polymeric composition of a selection of MPs from different locations and of different sizes and shapes were determined using a micro-Raman spectrometer (LabRAM HR, Horiba, Japan) with a laser of 785 nm, a Raman shift of 400–1800 cm^−1^ and acquisition times between 20 and 30 s. We used a microscope, needle, and tweezers to transfer MPs onto a conductive copper adhesive. To identify the polymers present, we utilized a tool called Open Specy (https://openanalysis.org/openspecy/) (accessed on 15 December 2022). This online, open-access database was developed by Cowger et al. [26] and enables users to compare spectra for material identification.

#### 2.3.3. Quality Assurance and Quality Control

A plastic avoidance procedure was taken prior to sampling, as all sampling containers and tools were washed with water, previously filtered through a white-gridded mixed cellulose ester membrane filter with a diameter of 47 mm and pore size of 0.45-μm (Frisenette, Denmark) and sealed. A blank sample procedure was developed to estimate the amount of contamination caused by the experiment to prevent sampling and analysis errors. It was confirmed that the blank samples were free of MP pollution. In addition, we used 100% cotton clothing and glass laboratory materials, wrapped materials immediately after treatments, and rinsed and cleaned all instruments prior to conducting laboratory analysis in the laboratory. To ensure that no microparticles remained in the solutions, all chemical solutions were filtered with sterile filter paper. A hydrogen peroxide test was provided for sample preparation [27]. In addition, SPSS Statistics 23.0 software was used for data analysis. The Kruskal-Wallis test was applied to the data on MPs occurrence and distribution in surface freshwater and wastewaters of Latvia and Lithuania to determine if there were statistically significant differences between four groups: summer samples in Latvia (SLV), winter samples in Latvia (WLV), summer samples in Lithuania (SLT), and winter samples in Lithuania (WLT) (WLT). At a confidence level of 95%, the test statistic value of 0.466 indicated that there was no significant difference in the median levels of MPs between the groups.

## 3. Results

### 3.1. Optical Microscopy Results

A comprehensive analysis of the presence and abundance of MPs in various water samples was conducted. As detailed in Tables S2 and S3, 103 particles were detected and recorded in total (Tables S2 and S3). It was determined that the average MP particle concentration in the influent was 5.00 ± 5.35 particles/L; in the effluent, it was 28.33 ± 23.17 particles/L, upstream it was 24.29 ± 31.55 particles/L, and downstream it was 15.00 ± 10.00 particles/L. It has been determined that the average concentration of MP particles in the cities of Liepaja, Daugavpils, Klaipeda, and Siauliai varies seasonally. In the summer, the MP particle concentration in Liepaja is 12.5 ± 11.90 particles/L; in Daugavpils, it is 28.75 ± 22.87 particles/L; in Klaipeda, it is 13.75 ± 11.09 particles/L, and in Siauliai, it is 27.5 ± 19.36 particles/L. In the winter, the MP particle concentration in Liepaja is 0.5 ± 3.54 particles/L; in Daugavpils, it is 0.25 ± 2.50 particles/L; in Klaipeda, it is 26.25 ± 42.70 particles/L, and in Siauliai, it is 11.25 ± 7.50 particles/L.

The average concentration of MPs ©n effluent samples is significantly higher than in influent samples, according to this study. The average concentration of MP particles was determined to be 28.33 ± 23.17 particles/L in the effluent and 5.00 ± 5.35 particles/L in the influent. Multiple factors may contribute to the increased concentration of MPs in effluent samples. One factor is that the water treatment process is ineffective at removing MPs. A source of MPs may also exist within the treatment facility, such as the breakdown of larger plastic items or the release of microfibers from textiles during laundering. In addition, the accumulation of MPs within the treatment facility over time may also have contributed to the increased concentration of MPs in effluent samples. This finding has significant implications for the management and treatment of wastewater, as it suggests that current treatment methods may not be fully effective at removing or reducing the abundance of MPs.

In 2021, samples from Lithuania fell within the smallest and biggest size category, with 11% and 47% of the samples averaging <0.25 mm and 1–5 mm, respectively. In Latvia and Lithuania, most samples were less than 1–5 mm in size. Moreover, the shape of MPs was evaluated. Fiber-shaped particles made up 95% of the samples from Lithuania and 5% of fragment shapes. Similarly, Latvia samples were primarily composed of fiber-shaped particles. Most of the particles found in Latvian samples were blue and black (61% and 36%) (Figure S2).

### 3.2. Micro-Raman Spectroscopy Results

A micro-Raman spectroscopy analysis was conducted on nine samples, representing 28% of the total sample population. These results provide important evidence for further investigation into the structure and composition of these samples.

**Figure 2:**
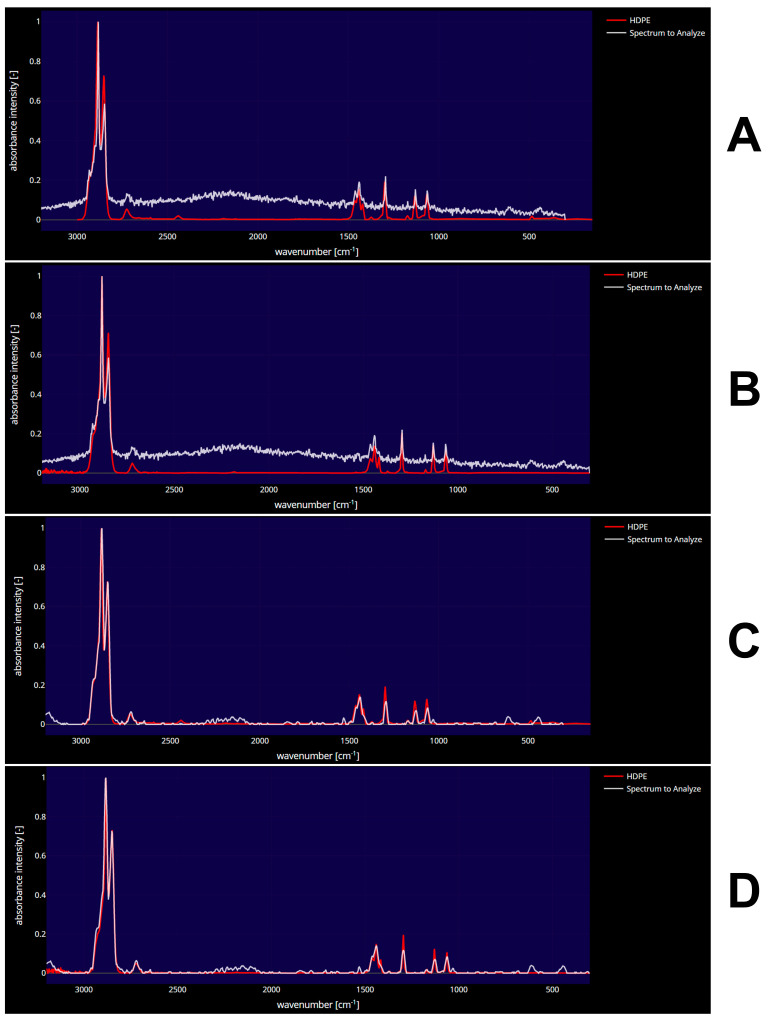
High-Density Polyethylene (HDPE) micro-Raman spectrum comparisons: (**A**) upstream in Klaipeda; (**B**) wastewater influent in Klaipeda; (**C**) wastewater effluent in Klaipeda; and (**D**) downstream in Klaipeda.

Figure 2 depicts the micro-Raman spectra of HDPE MPs in Klaipeda’s upstream water, wastewater influent, wastewater effluent, and downstream water samples. The characteristic peaks at 1002 cm^−1^, 1160 cm^−1^, and 1450 cm^−1^ in the micro-Raman spectra of HDPE MPs are attributed to the CH_2 bending mode, CH_2 wagging mode, and CH_2 rocking mode, respectively. The peak at 1694 cm^−1^ is due to the C = O stretching mode, which likely originates from an adhesive or coating material on the MP's surface. The similar intensities and peak positions of the spectra in panels (A) and (B) indicate that the concentration and composition of HDPE MPs in upstream and influent water are comparable. However, the spectra in panels (C) and (D) have lower intensities and peak shifts than those in panels (A) and (B), indicating that the concentration and composition of HDPE MPs in the upstream and wastewater influent are different from those in the effluent and downstream. This figure indicates that HDPE MPs are present in Klaipeda’s surface freshwater and wastewater and that their concentration and composition are influenced by wastewater treatment processes.

The results of the micro-Raman analysis of selected MPs indicated that there were five types of polymers. The most commonly encountered polymers among the fibers analyzed were polyethylene terephthalate (PET) (33%) and polyvinyl chloride (PVC) (33%), with lower percentages of nylon (NL) (12%), polyester (PS) (11%), and HDPE (11%) also detected (Figure S1). For instance, Figure 2 compares the micro-Raman spectra of HDPE MPs in surface freshwater and wastewater in Klaipeda, Lithuania. These comparisons shed light on the occurrence and distribution of HDPE MPs in surface freshwater and wastewater. Spectra from four distinct locations were analyzed and compared: upstream, wastewater influent, wastewater effluent, and downstream. The results indicate that all spectra exhibit an HDPE-specific peak. However, the intensity of the HDPE peak differs between spectra, indicating that the concentration of HDPE MPs in the various locations differs. The downstream and effluent spectrum has the lowest intensity, whereas the upstream and influent spectrum has greater intensity, indicating a greater concentration of HDPE MPs in the wastewater. Overall, the comparisons of micro-Raman spectra shown in Figure 2 demonstrate the widespread presence of HDPE MPs in surface freshwater and wastewater in Klaipeda. In addition, the results suggest that wastewater treatment processes may not be able to completely remove HDPE MPs from wastewater before it is discharged into the environment. Most of the particles analyzed via micro-Raman analysis were white (45%) or blue (33%). Black and red had the lowest rate among colors, with 11% each. This suggests that WWTPs may not be removing all pollutant particulates from the water, which could lead to adverse environmental impacts. In order to improve water quality, it is important to identify the sources of these pollutants and to implement measures that will reduce their presence in the water. The results of the micro-Raman analysis revealed that the vast majority of pollutants were between 1–5 mm (33%) and 0.5–1 mm (33%). A significantly smaller percentage of pollutants were between 0.25–0.5 mm (22%), while an even smaller percentage was below 0.25 mm (12%). These results suggest that there are a variety of pollutant sizes present in the surface water, which could have considerable impacts on water quality and the environment. Moreover, the shape of MPs was evaluated, and all of the particles were fiber-shaped. This is an indicator of the potential sources of the MPs in materials, such as synthetic textiles, which have been identified as potential sources of these contaminants. Additionally, the evaluation of MPs allowed for a comparison between the cities regarding their wastewater treatment efficiency. The results from this comparison will be used to guide further research into wastewater treatment and its potential effects on water quality.

## 4. Discussion

### 4.1. Study Limitations

This study has several limitations that should be noted. First, there were relatively few samples collected, which might limit the generalizability of the findings. In addition, the study concludes that municipal wastewater from catchment areas was the primary source of MP contamination in the surface water and wastewater of Latvia and Lithuania and suggests implementing pollution-reduction strategies, such as raising awareness, installing more high-tech WWTPs, and reducing plastic use. However, the study has a limited sample size and limited sampling periods, and the results may not be representative of other regions or seasons.

### 4.2. Microplastic Comparison in Latvia, Lithuania, and Other Aquatic Environments

In Latvia and Lithuania, there has been limited research on the levels of MPs in surface water and wastewater. It is important to study these levels in order to better understand the occurrence and distribution of MPs in these countries and to identify potential sources of contamination. By comparing the concentrations of MPs in Latvian and Lithuanian surface water and wastewater to those in other countries, researchers can gain a better understanding of the global distribution of MPs and the potential impacts they may have on aquatic ecosystems (Table 1).

The detection of MPs In aquatic environments, such as surface water and wastewater, has been extensively documented in the scientific literature. For instance, MPs have been detected in aquatic environments, such as brackish water [12], surface water [9], wastewater influent [28], and wastewater effluent [29]. This study is the first to report the occurrence of MPs in surface water and wastewater in Latvia and Lithuania, where they were found to have various shapes, colors, and sizes and to be composed of a range of polymers. Surface water and wastewater contain polymers with toxic and carcinogenic properties that may originate from industrial discharges, agricultural runoff, and sewage. Certain plastics, such as PVC and PET, and synthetic rubbers, such as neoprene, are examples of toxic polymers that can be found in surface waters and wastewater. Here, MPs were identified as having several shapes (fibers and fragments), various colors (transparent, yellow, red, blue, and black), and sizes (<0.25, 0.25–0.5, 0.5–1, and 1–5). Additionally, PET, PVC, NL, and PS polymers were determined in the form of a variety of polymers in various colors, forms, and sizes, demonstrating the diversity of MPs sources. The diversity of MPs sources observed in this study suggests that they may be coming from a variety of sources, including ships, wind, urban wastewater, and hospital wastewater. It can be said that MPs found in surface water and wastewater samples are likely to have originated from ships [30], wind [31], urban wastewater [32], and hospital wastewater [33].

The number of MPs identified in 32 samples of influent, effluent, upstream, and downstream ranged from zero (not detected) in two samples to 11 in samples SLT6 and WLT3. Besides, RA analysis showed different polymers, namely PET, PVC, NL, and PS, in 9 samples. There is a lack of information about the concentration of MPs in Latvia and Lithuania, making comparing this value with literature concentrations of MPs in surface water and wastewater problematic. For instance, one study on MPs in Lithuania detected 2982  ±  54 particles/L in the influent and 1244  ±  21 particles/L in the effluent [34]. In another study, [9] found 4430 particles/L in the Gulf of Riga.

**Table 1:** The abundance of MPs found in surface water and wastewater from different locations in the world, with the most abundant morphology, color, and chemical composition.

Location	Abundance (Particles/L)	Shape	Size	Color	Polymer	Reference
Latvia	3.50 ± 2.38 ^1^	Fiber	<0.25 mm 0.5–1 mm 1–5 mm	Red Blue Black	PET PVC NL PS	This study
Lithuania	7.50 ± 6.45 ^1^	Fiber	<0.25 mm 0.25–0.5 mm 0.5–1 mm 1–5 mm	Red Blue Black	PET PVC NL PS	This study
China	10.5 ± 2.5 ^1^	Fragments	0.01–0.1 mm 0.1–1 mm 1–5 mm	Transparent White Blue Black Yellow	PE PS PP PVC	[35]
China	654 ^1^	Fibers Fragments	50–100 μm 100–200 μm 200–500 μm 500–5000 μm	-	-	[36]
Portugal	231 ^1^	Fragments Spherule Fibers	-	Black Blue Brown White	-	[37]
Iran	0.027 ± 0.042 ^1^	Spherule Fibers	0.05–0.5 mm 0.5–1 mm 1–2.5 mm 2.5–5 mm >5 mm	Red	-	[38]
Lithuania	33.75 ± 40.08 ^2^	Fiber Fragment	<0.25 mm 0.25–0.5 mm 0.5–1 mm 1–5 mm	Transparent Yellow Red Blue Black	PET PVC NL PS	This study
Latvia	11.67 ± 12.58 ^2^	Fiber	<0.25 mm 0.25–0.5 mm 0.5–1 mm 1–5 mm	Blue Black	PET PVC NL PS	This study
Lithuania	2982 ± 54 ^2^	Fiber Fragment Pellet	20–50 μm 50–100 µm 100–200 µm 200–500 µm 500–1000 µm	Black White Transparent Brown Yellow Blue Other	PET PS PP	[34]
Lithuania	1244 ± 21 ^2^	Fiber Fragment Pellet	20–50 μm 50–100 µm 100–200 µm 200–500 µm 500–1000 µm	Black White Transparent Brown Yellow Blue Other	PET PS PP	[34]

^1^ Surface water. ^2^ Wastewater. CE—Cellulose, ET—Ethylene, EVA—Poly (Ethylene Co Vinyl Acetate), HDPE—High—density polyethylene, NL—Nylon, PAA—Poly (Acrylic Acid), PE—Polyethylene, PEL—Poly (ether- urethane), PET—Polyethylene terephthalate, PP—Polypropylene, PS—Polystyrene.

### 4.3. Challenges and Potential Solutions for Removing Microplastics from Wastewater

Several filtration systems can be used to remove MPs from wastewater. These systems typically use physical or chemical processes to capture and remove MPs from the water. One type of filtration system that is commonly used to remove MPs from wastewater is a microfiltration system [39]. Another type of filtration system that can be used to remove MPs from wastewater is ozonation [23]. Other types of filtration systems that can be used to remove MPs from wastewater include gravity filters [40], sand filters [40], ultraviolet radiation [40], chlorination [40], advanced oxidation processes [41], and activated carbon filters [40]. Because MPs are so small and can easily pass through a variety of filters, there is no known filtering system that can remove 100% of MPs from wastewater. Efforts are ongoing to develop more effective methods for removing MPs from water, but it is currently impossible to eliminate them entirely from wastewater. 

### 4.4. Untested Hypotheses and Potential Avenues for Future Research

There are several hypotheses that remain untested and potential avenues for future research that have been identified in this study. The first step is to identify the sources of MPs in Latvian and Lithuanian surface freshwater and wastewater in order to develop effective mitigation strategies. Secondly, further research is needed to determine how MPs are transported in freshwater systems. The transport of MPs can be affected by factors such as water flow rate, temperature, and sedimentation. Thirdly, it is unclear what effect MPs will have on freshwater ecosystems. A comprehensive assessment of MPs' effects on aquatic organisms and the environment is essential. Fourthly, to comprehend the long-term consequences of MP pollution, it is necessary to investigate the fate and persistence of MPs in freshwater systems. Lastly, it is necessary to investigate the occurrence and distribution of MPs in groundwater systems in order to identify potential sources and pathways of MPs and their potential environmental impacts.

## 5. Conclusions

This study presents the first assessment of the abundance and physical and chemical characteristics of MP litter from 16 sampling points located on the influent, effluent, upstream, and downstream locations in Latvia and Lithuania on the Baltic Sea. For this study, optical microscopy and micro-Raman spectroscopy methods were chosen to analyze samples because optical microscopy can observe color, shape, and size, while micro-Raman can identify polymer types and identify small MPs with a size of 20 microns; the combination of optical microscopy and micro-Raman spectroscopy allows accurate quantification of MPs and polymeric recognition. Finally, MPs detected in surface water and wastewater were mainly polymeric structures with various shapes, sizes, and colors. The nature of the different MPs indicates that the majority were secondary in nature, probably originating from rivers near the sampling stations, which receive municipal and industrial wastewater. As a result of the chemical characterization, polymers were identified as being very common in household waste, demonstrating the source of MP pollution. In addition, surface water and wastewaters contain polymers with toxic and carcinogenic properties. Having these data to evaluate the pollution caused by MPs on a local scale is fundamentally essential. As well as identifying the primary sources of pollution, they will serve as a basis for identifying possible accumulation hotspots.

## Data Availability

The authors confirm that all data supporting the findings of this study are available from the corresponding author upon request.

## Supplementary Materials

The following supporting information can be downloaded at: https://www.mdpi.com/article/10.3390/toxics11040292/s1, Figure S1: Types of polymers detected by RS in 9 water samples in Latvia and Lithuania.; Figure S2: Images of MPs observed in Latvia and Lithuania; (A) black fibers with a length of 1.1 mm in Klaipeda upstream, (B) black fibers with a length of 0.7 mm in Klaipeda effluent, (C) black fibers with a length of 0.6 mm in Siauliai influent, (D) black fibers with a length of 1.06 mm and 1.08 mm in Siauliai effluent, (E) black fibers with a length of 1.09 mm in Liepaja upstream, (F) black fibers with a length of 0.9 mm in Liepaja downstream, (G) black fibers with a length of 2.06 mm and 0.6 mm in Daugavpils influent, (H) black fibers with a length of 1.08 mm and 0.6 mm in Daugavpils effluent.; Table S1: Sampling location and physicochemical properties of influent, effluent, upstream and downstream in Latvia and Lithuania.; Table S2: Size, shape, and color of MP particles in surface water and wastewater of Latvia.; Table S3: Size, shape, and color of MP particles in surface water and wastewater of Lithuania.
