# Supplementary material for: Assessing the Occurrence and Distribution of Microplastics in Surface Freshwater and Wastewaters of Latvia and Lithuania
Source: Toxics. 2023 Mar 23;11(4):292. doi: 10.3390/toxics11040292 (PMC10146209; doi:10.3390/toxics11040292)
Supplement: Supplementary file 1 [file toxics-11-00292-s001.zip › toxics-2239193-supplementary.pdf]

toxics MDPI

Supplementary Information (SI)

Assessing the Occurrence and Distribution of Microplastics in Surface Freshwater and Wastewaters of Latvia and Lithuania

Reza Pashaei [1,*], Viktorija Sabaliauskaitė [1], Sergej Suzdalev [1], Arūnas Balčiūnas [1], Ieva Putna-Nimane [2], Robert M. Rees [3] and Reda Dzingelevičienė [4]

[1] Marine Research Institute, Klaipeda University, 92294 Klaipeda, Lithuania
[2] Latvian Institute of Aquatic Ecology, 1007 Riga, Latvia
[3] Scotland's Rural College (SRUC), West Mains Road, Edinburgh EH9 3JG, UK
[4] Faculty of Health Sciences, Marine Research Institute, Klaipeda University, 92294 Klaipeda, Lithuania

* Correspondence: reza.pashaei@ku.lt

Table S1 Sampling location and physicochemical properties of influent, effluent, upstream and downstream in Latvia and Lithuania.

Location	Acronym (summer/winter)	Latitude and longitude	pH (summer/winter)	Temperature (°C) (summer/winter)	Dissolved oxygen (mg/L) (summer/winter)
Klaipeda WWTP inlet	SLT1/WLT1[1,2]	55.639695, 21.253412	7.20/7.00	9.20/4.50	-
Klaipeda WWTP outlet	SLT2/WLT2[1,2]	55.639695, 21.253412	8.08/7.70	10.00/4.70	-
Klaipeda strait upstream	SLT3/WLT3[1,2]	55.665355, 21.147542	8.60/-	24.00/1.80	8.30/13.20
Klaipeda strait downstream	SLT4/WLT4[1,2]	55.669792, 21.145073	8.70/-	24.40/3.20	8.60/12.90
Siauliai WWTP inlet	SLT5/WLT5[1,2]	56.006635, 23.296027	7.94/7.87	21.00/12.70	-/0.85
Siauliai WWTP outlet	SLT6/WLT6[1,2]	56.006635, 23.296027	7.78/7.75	18.00/8.70	-/8.50
Siauliai strait upstream (Kulpe River)	SLT7/WLT7[1,2]	55.982637, 23.327158	-	22.90/6.10	5.09/9.34
Siauliai strait downstream (Kulpe River)	SLT8/WLT8[1,2]	55.989392, 23.338797	-	22.50/6.30	6.64/9.50
Liepaja WWTP inlet	SLV1/WLV1[3,4]	56.5968824, 21.0192835	-	-	-
Liepaja WWTP outlet	SLV2/WLV2[3,4]	56.5962711, 21.0203724	-	-	-
Liepaja strait upstream (Baltic Sea)	SLV3/WLV3[3,4]	56.599066, 20.998	-	-	-
Liepaja strait downstream (Baltic Sea)	SLV4/WLV4[3,4]	56.599066, 20.998	-	-	-
Daugavpils WWTP inlet	SLV5/WLV5[3,4]	55.8763647, 26.5052368	-	-	-
Daugavpils WWTP outlet	SLV6/WLV6[3,4]	55.8774556, 26.5051402	-	-	-
Daugavpils strait upstream	SLV7/WLV7[3,4]	55.87223, 26.50251	7.41/7.58	16.40/0.90	3.17/10.30
Daugavpils strait downstream	SLV8/WLV8[3,4]	55.87695, 26.50243	7.27/7.61	17.80/4.10	4.09/10.60

[1]Summer samples in Lithuania (SLT)
[2]Winter samples in Lithuania (WLT)
[3]Summer samples in Latvia (SLV)
[4]Winter samples in Latvia (WLV)

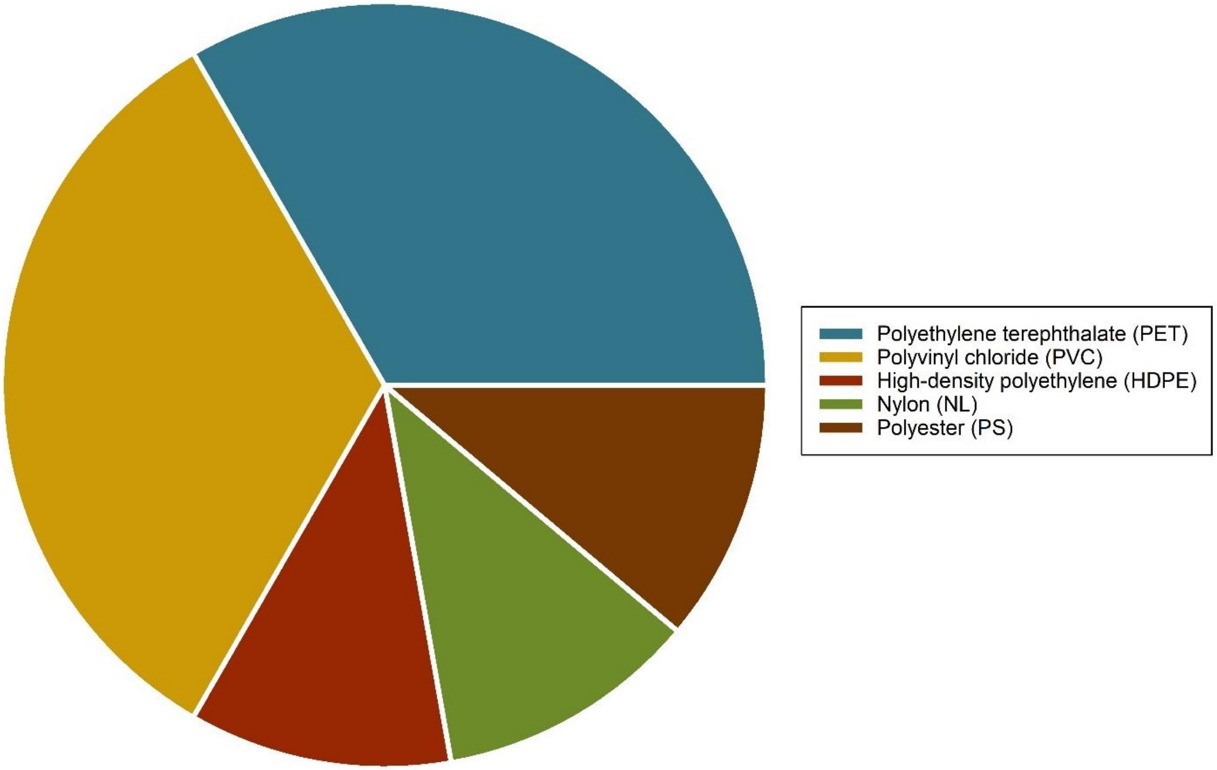

Figure S1. Types of polymers detected by RS in 9 water samples in Latvia and Lithuania.

Table S2 Size, shape, and color of MP particles in surface water and wastewater of Latvia.

Samples	Size (mm)				Shape		Color				
	<0.25	0.25-0.5	0.5-1	1-5	Fiber	Fragment	Transparent	Yellow	Red	Blue	Black
SLV1[1,6]	1	0	0	0	1	0	0	0	0	1	0
SLV2[1,6]	0	0	0	1	1	0	0	0	0	1	0
SLV3[1,5]	0	0	2	0	2	0	0	0	0	2	0
SLV4[1,5]	0	0	2	3	5	0	0	0	0	1	4
SLV5[1,6]	0	0	0	1	1	0	0	0	0	1	0
SLV6[1,6]	0	0	3	10	13	0	0	0	0	6	7
SLV7[1,5]	1	4	1	5	11	0	0	0	1	8	2
SLV8[1,5]	0	0	0	1	1	0	0	0	0	0	1
WLV1[2,6]	0	0	0	0	0	0	0	0	0	0	0
WLV2[2,6]	1	0	0	0	1	0	0	0	0	1	0
WLV3[2,5]	0	0	0	0	0	0	0	0	0	0	0
WLV4[2,5]	0	0	0	0	0	0	0	0	0	0	0
WLV5[2,6]	0	0	0	0	0	0	0	0	0	0	0
WLV6[2,6]	0	0	0	0	0	0	0	0	0	0	0
WLV7[2,5]	0	0	0	0	0	0	0	0	0	0	0
WLV8[2,5]	0	0	0	3	3	0	0	0	0	3	0

[1]Summer samples in Latvia (SLV)
[2]Winter samples in Latvia (WLV)
[3]Summer samples in Lithuania (SLT)
[4]Winter samples in Lithuania (WLT)
[5]Surface water
[6]Wastewater

Table S3 Size, shape, and color of MP particles in surface water and wastewater of Lithuania.

Samples	Size (mm)				Shape		Color				
	<0.25	0.25-0.5	0.5-1	1-5	Fiber	Fragment	Transparent	Yellow	Red	Blue	Black
SLT1[3,6]	0	3	0	0	2	1	0	0	1	1	1
SLT2[3,6]	0	1	2	2	5	0	0	1	2	2	0
SLT3[3,5]	0	6	0	0	6	0	0	0	1	4	1
SLT4[3,5]	0	0	1	1	2	0	0	0	0	1	1
SLT5[3,6]	0	0	1	0	1	0	0	0	0	1	0
SLT6[3,6]	2	2	0	7	11	0	2	2	1	3	3
SLT7[3,5]	1	0	1	5	7	0	0	0	1	5	1
SLT8[3,5]	0	0	0	4	4	0	0	0	0	1	3
WLT1[4,6]	0	0	0	0	0	0	0	0	0	0	0
WLT2[4,6]	4	0	0	0	2	2	0	0	0	3	1
WLT3[4,5]	0	0	7	4	11	0	0	0	0	5	6
WLT4[4,5]	0	0	1	0	1	0	0	0	0	1	0
WLT5[4,6]	0	0	0	3	3	0	0	0	0	3	0
WLT6[4,6]	0	0	1	3	4	0	0	0	0	2	2
WLT7[4,5]	0	1	0	0	1	0	0	0	0	1	0
WLT8[4,5]	0	0	0	1	1	0	0	0	0	1	0

[1]Summer samples in Latvia (SLV)
[2]Winter samples in Latvia (WLV)
[3]Summer samples in Lithuania (SLT)
[4]Winter samples in Lithuania (WLT)
[5]Surface water
[6]Wastewater

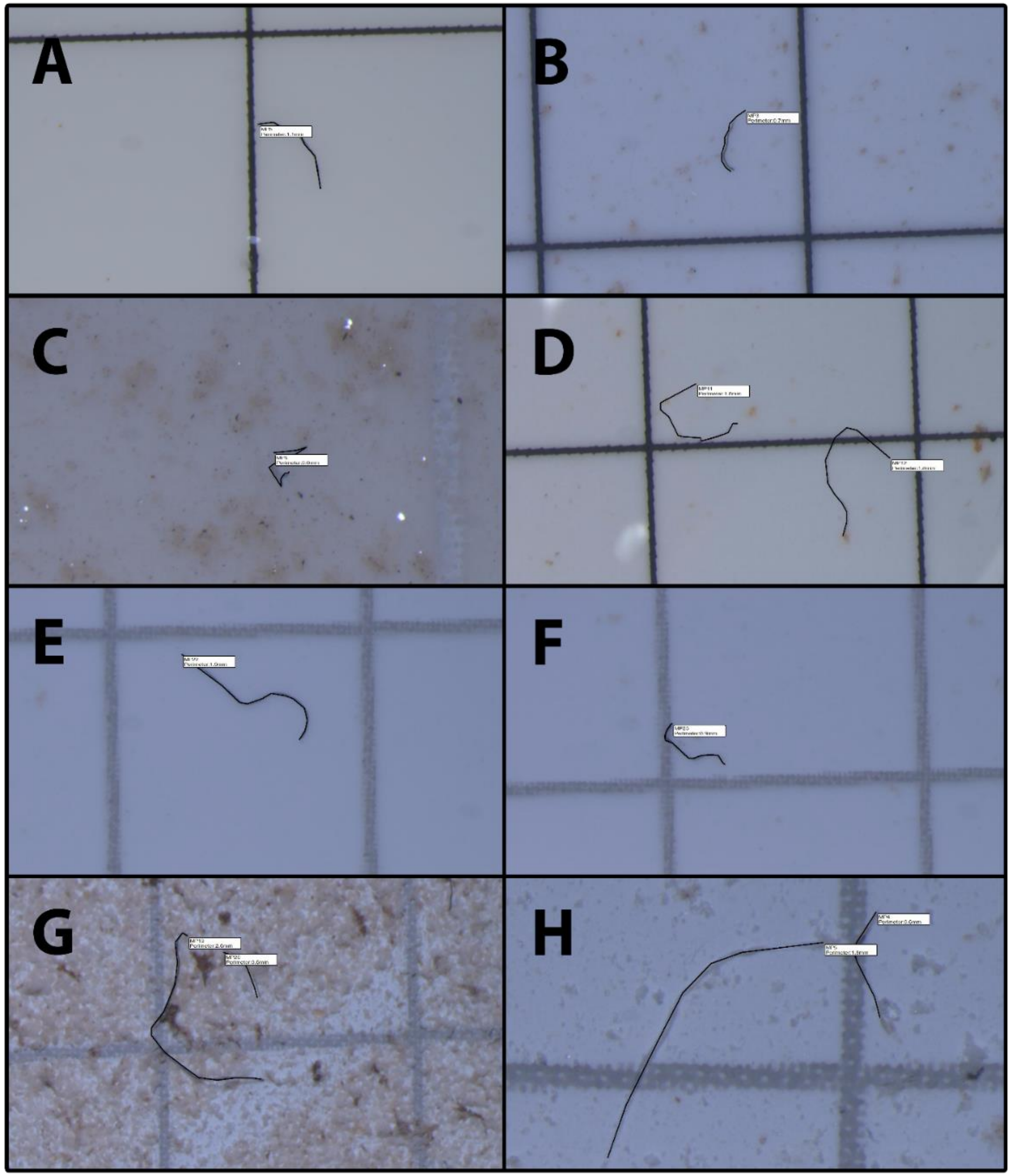
A
B
C
D
E
F
G
H

Figure S2. Images of MPs observed in Latvia and Lithuania; (A) black fibers with a length of 1.1 mm in Klaipeda upstream, (B) black fibers with a length of 0.7 mm in Klaipeda effluent, (C) black fibers with a length of 0.6 mm in Siauliai influent, (D) black fibers with a length of 1.06 mm and 1.08 mm in Siauliai effluent, (E) black fibers with a length of 1.09 mm in Liepaja upstream, (F) black fibers with a length of 0.9 mm in Liepaja downstream, (G) black fibers with a length of 2.06 mm and 0.6 mm in Daugavpils influent, (H) black fibers with a length of 1.08 mm and 0.6 mm in Daugavpils effluent.
